# Assessment of practice of Covid-19 preventive measures and associated factors among residents in Southern, Ethiopia

**DOI:** 10.1371/journal.pone.0261186

**Published:** 2021-12-10

**Authors:** Abdene Weya Kaso, Habtamu Endashaw Hareru, Gebi Agero, Zemachu Ashuro

**Affiliations:** 1 School of Public Health, College of Medicine and Health Science, Dilla University, Dilla, Ethiopia; 2 Department of Public Health, College of Health Science, Arsi University, Assela, Ethiopia; The Chinese University of Hong Kong, HONG KONG

## Abstract

**Introduction:**

Coronavirus (Covid-19) is a respiratory disease mostly affecting old aged and those with comorbidities. Due to resource constraints in developing countries, control of Covid-19 was based on preventive measures. However, there is variation in adherence to these precautionary measures from place to place among communities. Therefore, this study assessed the practice of Covid-19 preventive measures and associated factors among residents of Southern, Ethiopia.

**Methods:**

A community-based cross-sectional study was employed on residents of Southern, Ethiopia. Interviewer administered questionnaire was used to collect data from households through systematic random sampling methods. Data was entered, coded, and analyzed using STATA version 16. Logistic regression analysis was used to explore the association between outcome variables and predictor variables. Finally, the interpretation of Adjusted Odds ratio (AOR) with 95% Confidence Interval (CI) and p-value was done for statistically significant factors of Covid-19 preventive measures practice.

**Results:**

The proportion of residents who had good practice of Covid-19 preventive measures was 31.3% (95% CI: 26.5, 36.1). Out of 364 residents, 264 (72.5%) used facemasks, 218 (59.9%) washed their hands frequently, 167 (45.9%) practiced social distancing, 135 (37.1%) stayed at home, 75 (18.1%) avoided handshaking and 228 (62.6%) used hand sanitizer. Following government directions (AOR = 225; 95% CI: 68.6, 738), good knowledge about Covid-19 (AOR = 3.47; 95% CI: 1.12, 10.73), having access to water supply (AOR = 2.92; 95% CI: 1.05, 8.18), belief towards protectiveness of preventive measure (AOR = 3.53; 95% CI: 1.08, 11.61) and chronic illness (AOR = 5.09; 95% CI: 1.44, 17.96) were significantly associated with practice of Covid-19 preventive measures.

**Conclusion:**

In this study, the proportion of residents practicing Covid-19 preventive measures was low. Having comorbidity, following government directions, knowledge about Covid-19 and access to water supply were significantly associated with Covid-19 preventive measures practice. Therefore, government and all concerned stakeholders should increase the accessibility of infrastructure and provide continuous awareness creation campaigns regarding Covid-19 mode of transmission, sign and symptom, and protectiveness of Covid-19 preventive measures. Moreover, dissemination of teaching aids using local languages and close monitoring of community compliance to Covid-19 preventive measures is crucial.

## Background

Coronavirus disease (Covid-19) is a new life-threatening pandemic affecting mainly old aged people and those with comorbidity [1, 2]. As the virus has the potency to spread swiftly, it was declared a pandemic by the World Health Organization (WHO) on March 11, 2020 [3].

By November 4, 2021, Covid-19 cases reached 247,472,724 with 5,012,337 deaths globally. Until November 4, 2021, 6,092,498 confirmed cases, with 150,428 deaths were reported from African countries including Ethiopia. Ethiopia reported its first Covid-19 case on 13 March 2020. Ethiopia has reported 366,424 cases and 6,509 deaths until 4^th^ November 2021 because of this pandemic [4, 5]. Covid-19 spreads through millions of droplets released and patients infected with this virus have a high fever, dry cough, difficulty in breathing, chest pain, and sore throat. Around 5% of patients with Covid-19 disease, and 20% of patients hospitalized due to virus, experience worsening health conditions necessitating admission to the intensive care unit [6]. The occurrence of the Covid-19 pandemic has a tremendous impact on countries’ economies and brings financial hardship, especially in Low to middle-income countries (LMIC) [7]. Thus, countries have closed schools and Universities, ordered obligatory quarantine, restricted public gatherings like mass sport, meetings, and religious festivals to reduce the spread of the virus.

However, the effectiveness of the Covid-19 preventive methods needs communities’ adherence and belief in the protectiveness of the measure as a whole. Though WHO recommended physical distancing, wearing a facemask, and using hand sanitizer, there is variation in compliance to the preventive measures from country to country [3]. For instance, in Iran and China, around 89% and 96.4% of the residents adhered to Covid-19 preventive measures appropriately [8, 9]. But in Ghana, the proportion of utilization of facemasks is 31.5%, frequent hand washing is 49.5%, and social distancing is 46.2% among students [10]. Moreover, the proportion of Antenatal Care mothers who practiced wearing a facemask, hand sanitizer, and physical distancing in Ghana was 18.0%, 31.7%, and 22.0% respectively [11]. Even though mass community campaigns were conducted related to Covid-19, there is poor adherence to preventive measures in Ethiopia [12]. The prevalence of utilization of Covid-19 preventive measure was (49%) in Addis Ababa [13], (12.3%) in Dirashe district [14], (38.73%) in Gondar zone [15], (40.7%) in Dire Dawa city [16] and (44.4%) in Dessie, Northeast Ethiopia [17]. Studies also indicated that the community level of practicing Covid-19 preventive measures varies with sociodemographic factors, health-related factors, behavioural related factors, supply-side factors, ability to afford Personnel Protective Equipment (PPE), and beliefs toward the protectiveness of the PPE [18–21]. In resource-constrained countries, prevention and control of Covid-19 were based on preventive measures. Thus, community adherence to precautionary measures is needed to tackle the transmission of the virus [22]. Although information concerning Covid-19 preventive measures was disseminated through different ways and proclamation was declared to reduce the spread of Covid-19 infection in Ethiopia, the Covid-19 preventive measures were not well practiced on the ground. But, the effective management and control of infection needs evidence that shows the level of the Covid-19 prevention strategies at the community level. This helps policymakers, health providers, and concerned stakeholders to design appropriate strategies. Therefore, the aim of this study was to determine the resident level of practicing Covid-19 preventive measures and associated factors in Southern, Ethiopia.

## Method and materials

### Study area

The study was conducted in Wenago town, Gedeo zone, Southern Ethiopia. The altitude of this zone ranges from 1268 meters above sea level in the vicinity of Lake Abaya to 2993 meters at Haro Wolabu Pond. The zone has a total population of 1,247,812 (i.e., 624,931 are men and 622,881 women) with an area of 1,210.89 square kilometres. Gedeo zone is one of the highly populated areas with a population density of 699.84 according to the 2007 Ethiopian central statistical agency report. The economic activities of this area mainly depend on cash-crop like coffee. Wenago town is the administrative town of Wenago district. It has a population size of 15,239 with 3,110 households. It is 372km far from Addis Ababa and 12km from zonal town, Dilla town. The town has four kebele (smallest administrative units), one health center, and two health posts.

### Study design, population, and period

A Community based cross-sectional study design was carried out among households living in Wenago town from March 1–10, 2021.

### Sample size and sampling techniques

A single population formula was used to calculate sample size with the assumption of 49% Covid-19 preventive measures practice [13], 95% CI, and 5% margin of error. Since the total households in the town were less than 10,000, the correction formula was used to calculate the final sample size. By adding a 10% non-response rate the final sample size was 376. Two kebele (the smallest administrative unit) were randomly selected from four kebele in Wenago town. Then the sample was proportionally allocated to each kebele and a systematic random sampling technique was used to collect data from households. In the study, household heads (i.e., mother/father) or adults aged 18 and above years were included. But, the primary consideration was provided to the head of the households for the interview from the selected families. If the household head was not available during the data collection period, we interviewed adults aged 18 and above years old. In the case where more than one adult presented in that household, we used the lottery method to select one household member. For a household where no members were present, the next household was considered. Those who lived in the town for at least 6 months were included. The first household was recruited by lottery method and an interview was conducted at every 6^th^ interval until the sample size was fulfilled.

### Study variables

The primary outcome variable of the study was the practice of Covid-19 preventive measures. Sociodemographic factors, health-related factors, knowledge towards Covid-19, attitude towards protectiveness of the preventive measures, accessibility to preventive equipment, and acceptance of government recommendations were independent variables.

### Data collection procedure and quality assurance

The interviewer-administered structured questionnaire was developed after critical reviews of previously published articles on Covid-19 preventive practices [8, 14, 19, 23]. The questionnaire was prepared in English initially and the interview was made using the Amharic version. It was assessed for content validity with the view point of health experts. They were asked to indicate each item as essential or not essential and their overall score was in the acceptable range of the content validity index. Moreover, the questionnaire was pre-tested on 5% of the sample in Yirga Chafe town that has almost similar characteristics to the study area, and the amendment was done to ensure consistency. The data collection tool has sociodemographic data, health-related factors, Covid-19 knowledge, attitude, and prevention practice-related questions. Knowledge was measured using 12 items concerning clinical signs and symptoms, mode of transmission, treatment information, and the Covid-19 prevention method. Each item was measured using yes, no, or I don’t know questions. A yes answer was coded as one point whereas the no or I don’t know the answer was recoded as zero. We dichotomized the overall knowledge into poor or good knowledge and respondents who correctly answered above six items were considered to have good knowledge. The Cronbach alpha coefficient of the knowledge item was 0.82, indicating acceptable internal consistency. Moreover, the respondents’ Covid-19 preventive measures practice was assessed by six yes or no questions. These questions include frequent hand washing using soap and water, use of hand sanitizer, self-isolation during having signs and symptoms, use of facemasks, physical distancing, and avoiding handshaking as preventive measures. The participants’ practice was categorized into poor and good practice. Those respondents who correctly practiced four and more of the Covid-19 preventive measures were classified as good practice. Every day, the principal investigator monitored the completeness and quality of the data collected.

### Data processing and analysis

The collected data were entered, coded, cleaned, and analyzed using STATA Version 16. Continuous variables were summarized using mean, Inter Quartile Range (IQR), and Standard deviations (SD) whereas frequency and percentage for categorical variables were computed and presented in text, table, and graphs. Logistic regression analysis was performed to determine the relationship between explanatory variables and the outcome variables. Variables with a p-value below 0.25 were transferred into multivariate logistic regression analysis and a P-value less than 0.05 was declared to be statistically significant.

### Ethics approval and consent to participate

Ethical approval was obtained from the Institutional review board of Dilla University before the initiation of the study. Data collection permission was obtained from Wenago town administration bodies and study participants were informed about the benefit of participating in the study. As the time was under strict emergency state, to minimize the contacts and keep the social distancing, oral informed consent was acceptable and approved by the IRB of Dilla University, College of Medicine and Health Science. To safeguard the confidentiality of information, unique codes were provided on the questionnaire during the interview.

## Result

### Socio-demographic and economic characteristics of the respondents

The overall response rate of this study was 96.8%. In this study, the majority of the participants 178 (48.9%) were in the age group of 18–29 years and 193 (53%) of them were males. About 224 (61.5%) of respondents were married while nearly 209 (57.4%) of the respondents had primary and above education status. More than half 195 (53.6%) of the residents had a family size of greater than four with a mean family size of five [IQR: 4, 7] (Table 1).

**Table 1:** Sociodemographic and economic characteristics of the residents, Southern, Ethiopia, 2021.

Variable	Categories	Frequency (%)
Sex	Male	193 (53)
	Female	171 (47)
Age	18–29 years	178 (48.9)
	30–39 years	115 (31.59)
	40–49 years	43 (11.81)
	50 and above years	28 (7.69)
Religion	Orthodox	132 (36.3)
	Muslim	42 (11.5)
	Protestant	190 (52.2)
Marital Status	Single	113 (31)
	Married	224 (61.5)
	Divorced	27 (7.4)
Education	Non-formal	34 (9.3)
	Primary (1–8)	121 (33.2)
	High school	118 (32.4)
	College and above	91 (25)
Occupational status	Government employer	92 (25.3)
	Private worker	63 (17.3)
	Farmer	61 (16.8)
	Merchant	64 (17.6)
	Other	84 (23.1)
Income	<1000 ETB	138 (37.9)
	1000–1999 ETB	100 (27.5)
	2000–4000 ETB	81 (22.3)
	>4000 ETB	45 (12.4)
Family size	≤ 4 members	169 (46.4)
	>4 members	195 (53.6)

### Participants source of information and perception towards Covid-19 preventive measures

Of 364 residents, 185 (50.8%) heard about Covid-19 from television whereas 59 (16.2%) get information from government campaigns. Moreover, 52 (14.3%) of the residents believe that Covid-19 preventive measures tend to protect them from getting viruses. Around 125 (34.3%) of respondents feel insecure if they don’t practice the preventive measures and someone stands around them (Table 2).

**Table 2:** Information source and perception towards Covid-19 preventive measures among residents of Southern, Ethiopia, 2021.

Information source/perception Question	Category	Frequency (%)
Have you Heard about Covid-19?	Yes	364 (100)
Source of Information	Radio	77 (21.2)
	Television	185 (50.8)
	Government	59 (16.2)
	Social media	43 (11.8)
Where do you go for treatment if you get Covid-19?	Health Facilities	316 (86.8)
	Traditional Healer	11 (3)
	Self-treatment	18 (4.9)
	other	19 (5.2)
Do you Believe the Preventive method is protective?	Yes	52 (14.3)
	No	312 (85.7)
Do you feel insecure if someone stands around you?	yes	125 (34.3)
	No	239 (65.7)
Do you listen and follow government regulations?	Yes	108 (29.7)
	No	256 (70.3)

#### Individuals knowledge about Covid-19

Among four dimensions of Covid-19 knowledge assessed, 243 (66.7%), 74 (20.3%), 260 (71.4%), and 334 (91.8%) of respondents provided the correct answers for signs and symptoms, mode of transmission, treatment-related information, and prevention methods dimensions respectively. Regarding overall Covid-19 knowledge, 130 (20.5%) of the study subjects have good knowledge whereas 504 (79.5%) of them have poor knowledge about Covid-19 (Table 3).

**Table 3:** Knowledge about Covid-19 among respondents of Wenago town, Gedeo zone, Ethiopia, 2021.

Categories	Items	Yes (%)	No (%)	I don’t know (%)
**Signs and symptoms**	Dry cough	281 (77.2)	80 (22)	3 (0.8)
	Fatigue	270 (74.2)	79 (21.7)	15 (4.1)
	Head ache	264 (72.5)	100 (27.5)	0
	Sore throat	266 (73.1)	89 (24.5)	9 (2.5)
	Shortness of breath	157 (43.1)	161 (44.2)	46 (12.7)
	Fever	218 (59.9)	134 (36.8)	12 (3.3)
**Mode of transmission**	Covid-19 transmitted through droplet	75 (20.6)	252 (69.2)	37 (10.2)
	Covid-19 transmitted by contact with materials contaminated with a virus?	73 (20.1)	278 (76.4)	13(3.6)
**Treatment information**	There is no effective antibiotic treatment available for Covid-19?	293 (80.5)	68 (18.7)	3 (0.8)
	Does supportive care assist recovery from Covid-19 infection?	215 (59.1)	136 (37.4)	13 (3.6)
	Covid-19 is curable if treated and managed well?	272 (74.7)	83 (22.8)	9 (2.5)
**Prevention method**	Frequent hand washing or using hand sanitizer and facemask reduces Covid-19 infection?	334 (91.8)	30 (8.2)	0
Overall Knowledge	Poor knowledge	140 (38.5)		
	Good Knowledge	224 (61.5)		

### Magnitude of practicing Covid-19 preventive measures

**Fig 1:**
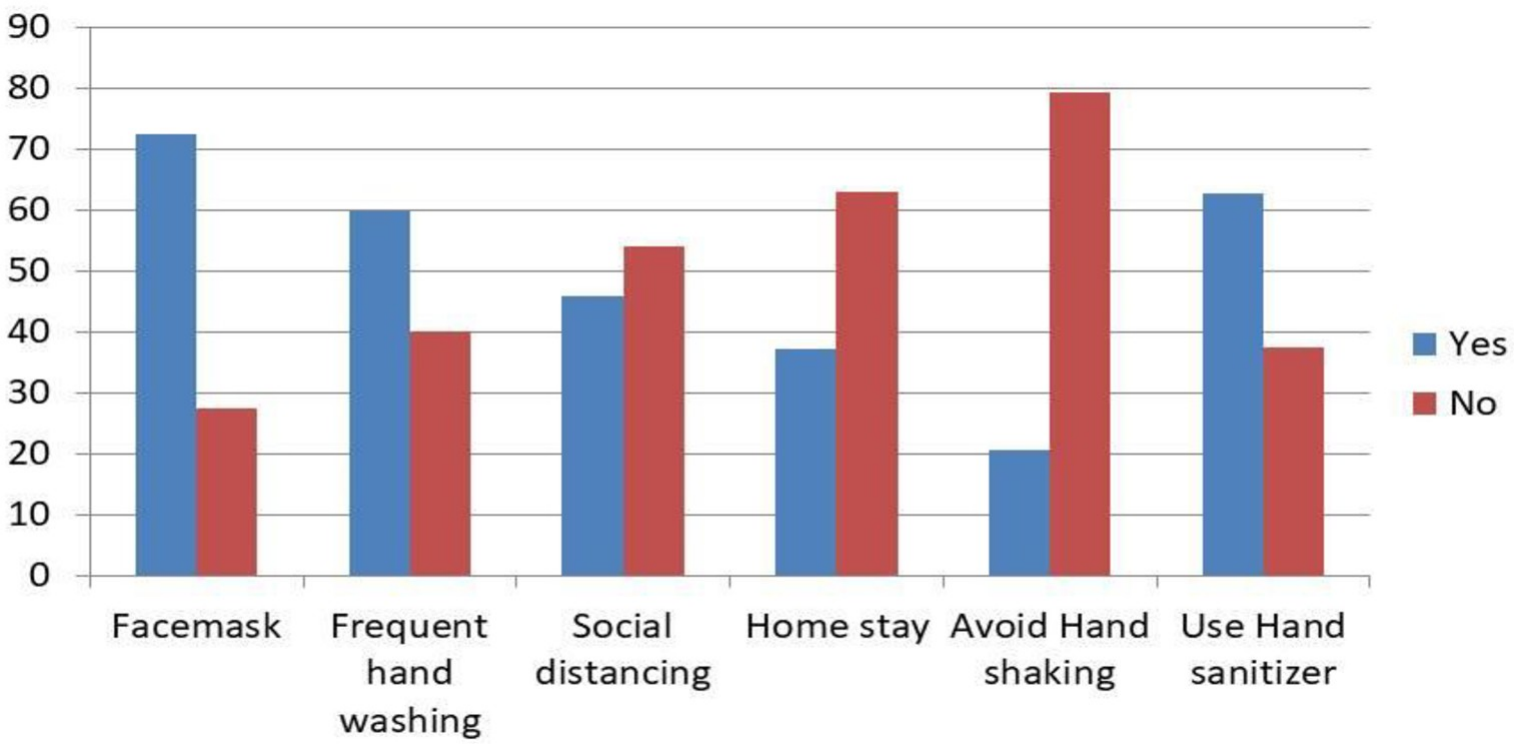
Practice of the Covid-19 preventive measures among residents of Southern, Ethiopia, 2021.

**Fig 2:**
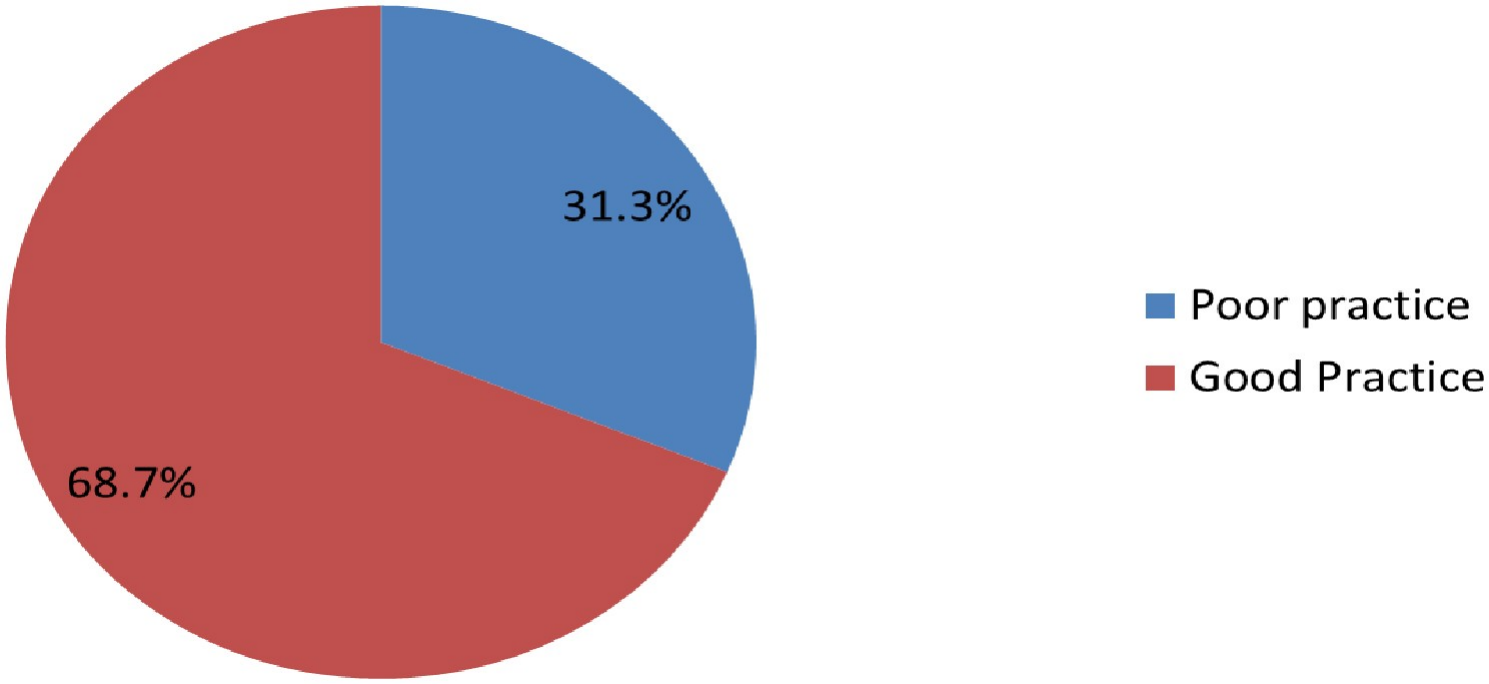
Magnitude of Covid-19 preventive measures practice among residents of Southern, Ethiopia, 2021.

One hundred fourteen (31.3%, 95% CI: 26.5, 36.1) of residents had good practice whereas 250 (68.7%) of respondents had the poor practice of Covid-19 preventive measures. Regarding respondents practice towards the Covid-19 preventive measures, 264 (72.5%) used facemasks, 218 (59.9%) frequently washed their hands with soap, 167 (45.9%) kept social distancing, 135 (37.1%) stayed at home, 75 (20.6%) avoided handshaking while 228 (62.6%) disinfected their hand with alcohol-based sanitizer (Figs 1 and 2).

### Factors associated with the practice of Covid-19 preventive measures

In bivariate logistic analysis variables such as sex, access to facemask, water supply, and hand sanitizer, age, following government directions, knowledge about Covid-19, perception toward protectiveness of the preventive measures, family size, and having chronic illness were significantly associated with the practice of Covid-19 preventive methods. However, in the multivariate logistic regression model following Government directions, knowledge about Covid-19, access to water supply, belief in the protectiveness of preventive measures, and having chronic illness were found to be statistically significant factors. The odds of practicing Covid-19 preventive measures in those who strictly follow government directions was 225 times higher than those who do not (AOR = 225; 95% CI: 68.6, 738). Moreover, those who had good knowledge about Covid-19 were 3.47 times more likely to practice Covid-19 preventive measures compared to their counterpart (AOR = 3.47; 95% CI: 1.12, 10.73). In the same way, the participants who had access to water supply were 2.92 times more likely to practice Covid-19 preventive measures than those don’t have (AOR = 2.92; 95% CI: 1.05, 8.18). Furthermore, the odds of practicing Covid-19 preventive measures among respondents with chronic illness were 5.09 higher than those without underlying diseases (AOR = 5.09; 95% CI: 1.44, 17.96). Besides this, individuals who believed in the protectiveness of Covid-19 preventive measures were 3.53 times more likely to practice Covid-19 preventive measures than those who do not (AOR = 3.53; 95% CI: 1.08, 11.61) (Table 4).

**Table 4:** Multivariate logistic analysis of factors associated with Covid-19 preventive measures practice among residents of Southern, Ethiopia, 2021.

Categories		Practice		COR(95%CI)	AOR(95%CI)
		Good (%)	Poor (%)		
Age	18–29 year	47 (26.4)	131 (73.6)	1	1
	30–39 years	42 (63.48)	73 (36.52)	1.60 (0.97, 2.66)	0.82 (0.28, 2.43)
	40–49 years	13 (30.23)	30 (69.77)	1.21 (0.58, 2.51)	2.06 (0.44, 9.61)
	≥50 years	12 (42.86)	16 (57.14)	2.09 (0.92, 4.74)	4.04 (0.72, 22.68)
**Sex**	Female	60 (35.09)	111 (64.91)	1.39 (0.89, 2.17)	2.49 (0.97, 6.36)
	Male	54 (27.98)	139 (72.02)	1	1
**Marital**	Single	33 (29.2)	80 (70.8)	1	1
	Married	69 (30.8)	115 (69.2)	1.08 (0.66, 1.77)	0.63 (0.21, 1.91)
	Divorced	12 (44.44)	15 (55.56)	1.94 (0.82, 4.59)	0.08 (0.02, 0.50)*
**Education**	Non-formal	14 (41.18)	20 (58.82)	1	1
	Primary	41 (33.9)	80 (66.1)	0.73 (0.34, 1.60)	0.51 (0.10, 2.56)
	High school	36 (28)	81 (72)	0.56 (0.25, 1.23)	0.62 (0.12, 3.40)
	College and above	20 (28.6)	62 (71.4)	0.57 (0.25, 1.30)	0.28 (0.02, 3.34)
**Occupation**	Government	28 (30.4)	64 (69.6)	1	1
	Private	21 (33.3)	42 (66.7)	1.14 (0.58, 2.27)	1.08 (0.22, 5.36)
	Farmer	20 (32.8)	41 (67.2)	1.11 (0.56, 2.23)	0.78 (0.10, 5.94)
	Merchant	20 (31.2)	44 (68.8)	1.04 (0.52, 2.07)	0.72 (0.12, 4.25)
	Other ^a^	25 (29.8)	59 (70.2)	0.97 (0.51, 1.85)	0.56 (0.08, 3.83)
**Income**	<1000 birr	47 (34.1)	91 (65.9)	1	1
	1000–1999 birr	30 (30)	70 (70)	1.14 (0.58, 2.27)	1.49 (0.39, 5.67)
	2000–4000 birr	24 (29.6)	57 (70.4)	1.11 (0.56, 2.23)	2.28 (0.41, 12.53)
	≥4000 birr	13 (28.9)	32 (71.1)	1.04 (0.52, 2.07)	2.71 (0.30, 24.19)
**Family size**	≤4	48 (28.4)	121 (71.6)	1	1
	>4	66 (33.9)	129 (66.1)	1.14 (0.58, 2.27)	1.08 (0.43, 2.71)
**Comorbidity**	Yes	26 (55.3)	21 (44.7)	3.22 (1.72, 6.02)	5.09 (1.44, 17.96)*
	No	88 (27.8)	229 (72.2)	1	1
**Follow government direction**	Yes	96 (88.9)	12 (11.1)	106 (49.1, 228)	225 (68.6, 738)*
	no	18 (7)	238 (93)	1	1
**Access to water**	Yes	96 (39.5)	147 (60.5)	3.74 (2.13, 6.56)	2.92 (1.05, 8.18)*
	No	18 (14.9)	103 (85.1)	1	1
**Overall Knowledge**	Poor	11 (7.9)	129 (92.1)	1	**1**
	Good	103 (46)	121 (54)	9.98 (5.11, 19.5)	3.47 (1.12, 10.73)*
**Believe utilization is protective**	yes	20 (38.5)	32 (61.5)	1.45 (0.79, 2.66)	3.53 (1.08, 11.61)*
	No	94 (30.1)	218 (69.9)	1	1
**Access to Facemask**	yes	104 (38.2)	168 (61.8)	5.08 (2.52, 10.3)	1.98 (0.54, 7.25)
	no	10 (10.9)	82 (89.1)	1	1
**Access to hand Sanitizer**	Yes	80 (33.1)	162 (66.9)	1.28 (0.79, 2.06)	1.8 (0.7, 4.64)
	No	34 (27.9)	88 (72.1)	1	1

Notes:

*Significant at p-value < 0.05;

^a^ Student, housewife, daily labour.

## Discussion

The Covid-19 virus was a pandemic devastated within a short period to many parts of the world. Effective and efficient controlling of the pandemic requires strict adherence to precautionary measures. Therefore, this study assessed the community level of practicing of Covid-19 preventive measures among residents in Wenago town, Ethiopia. Our study found that 31.3% of residents had good practice of Covid-19 preventive measures. This finding was lower than findings from Saudi Arabia (81%) [24], China (98%) [9], and Addis Ababa (51%) [13]. However, it is greater than the study findings from Dirashe District, Southern Ethiopia (12.3%) [14]. The variation is explained by the difference in the study participant, study period, and cut-off point used to dichotomize preventive measures practice to good or poor practice.

In this study, around 72.5% of the residents used facemasks and 59.9% practiced hand washing with soap, which is not much different from that of studies from Gedeo Zone (58.3%) [25], Dire Dawa (62.4%) [16], and Dirashe district (65.8%) [14]. But it is higher than that of study from Ghana (31.7%) [11] and lower than the finding from Addis Ababa (85%) [13] and Dessie (79.2%) [26]. The reasons for inconsistency could be the differences in the level of respondents’ awareness and belief toward protectiveness of Covid-19 preventive measure, time of studies, the number of Covid-19 cases reported and shortage of infrastructure as the Gedeo zone is densely populated.

We found that residents’ higher knowledge score is associated with higher Covid-19 preventive practices, a similar finding was reported between the knowledge about the disease and preventive practices from Bangladeshi [23], Ghana [10], Pakistan [27], Addis Ababa [13] and Debre Tabor [28]. This is might be due to the fact that those who had good knowledge about Covid-19 have adequate information about the consequence of the virus on their health, families, and overall activities if they get infected with the virus.

Our study found that strictly following government directions and residents’ belief towards protectiveness of preventive measures were significantly associated with good Covid-19 practice the preventive measures. This finding is similar to a multi-country study and a mixed approach study from Eastern Ethiopia that reported the same trends [12, 16]. In addition, studies revealed that the individual’s adoption of the precautionary measures depends on the individual’s belief towards the government directions, protectiveness, and effectiveness of Covid-19 preventive measures [12, 20, 21, 29, 30]. Besides this, those who had access to water supply were 2.92 times more likely to practice preventive measures compared to those who do not have. This is consistent with studies from Congo [31], Dire Dawa [16], and Amhara region, Ethiopia [26]. In Ethiopia, most of the residents had a shortage of adequate water supply which is the ultimate barrier to practicing frequent hand washing to reduce Covid-19 infections. Thus, participants who have a continuous water supply in their compound have the advantage of washing their hands with soap regularly compared with those who don’t have.

The study also points out that the odds of practicing Covid-19 preventive measures were 5.09 times higher among residents with chronic illnesses compared to those without underlying disease. A similar degree of findings was reported from studies in the Gedeo zone [25] and Amhara Region, Ethiopia [26] that revealed individuals with underlying health conditions were more likely to adhere to Covid-19 preventive strategies. This is most probably due to the fear of Covid-19 on their health as a result of information disseminated through Media about the impact of Covid-19 on individuals with chronic illnesses [32]. Even though our study used a validated questionnaire for data collection, it is not free from limitations. First, the cross-sectional nature of the study may not point out the temporal cause-effect relationship. Secondly, few community segments practice Covid-19 preventive measures only at public institutions due to fear of the government proclamation. Respondents sometimes overestimated their adherence to the preventive measures compared to the real data observed on the ground. Measuring the residents’ adherence with questionnaires only might influence the result of our study to some extent.

## Conclusion

The proportion of residents who had good practice of Covid-19 preventive measures was low. Having comorbidity, following government directions, belief toward protectiveness of preventive measures, knowledge about Covid-19, and access to water supply were significantly associated with Covid-19 preventive measures practice. Therefore, government and concerned stakeholders should work on the construction of extra infrastructure to address the issue of clean water accessibility and provide continuous awareness creation campaigns regarding Covid-19 mode of transmission, signs and symptoms, and protectiveness of Covid-19 preventive measures. Moreover, dissemination of teaching aids using local languages and close monitoring of community compliance to Covid-19 preventive measures is crucial.

## Supporting information

S1 Questionnaire (DOCX)

S2 Questionnaire (DOCX)

S1 Dataset (DTA)

## Abbreviations

AOR: Adjusted Odd Ratio
CI: Confidence Interval
COR: Crude Odd Ratio
LMIC: Low to Medium-Income Countries
OR: Odd Ratio
PPE: Personnel Protective Equipment
WHO: World Health Organization
