# Supplementary material for: Assessment of practice of Covid-19 preventive measures and associated factors among residents in Southern, Ethiopia
Source: PLoS One. 2021 Dec 10;16(12):e0261186. doi: 10.1371/journal.pone.0261186 (PMC8664224; doi:10.1371/journal.pone.0261186)
Supplement: S1 Questionnaire — (DOCX) [file pone.0261186.s001.docx]

**ዲላ የዩኒቨርሲቲ**

**የህክምና እና የጤና ሳይንስ ኮሌጅ**

**የህብረተሰብ ጤና ት/ቤት**

**ፍቃድ መጠየቂያ ቅፅ**

የተሳታፊዎች ስምምነት ከቃለ ምልልሱ በፊት የምረጋገጥበት፣ የ ኮቪድ-19 የመከላከያ እርምጃዎችን እና ተጓዳኝ ምክንያቶችን ፣ በደቡብ ክልል የጌዴኦ ዞን ፣ ወናጎ ከተማ 2020 ፣ እ.ኤ.አ፡፡

የቀበሌ ስም ________________________

መጠይቅ መታወቂያ ቁጥር ________________

መግቢያ - ስሜ _______________________ ስለ ኮቪድ-19 የመከላከያ እርምጃዎች ልምምድ እና ተጓዳኝ ምክንያቶች የወናጎ ነዋሪዎችን እያነጋገርኩ ነው። በጥናቱ ውስጥ ከተሳታፊዎች አንዱ ለመሆን ተመርጠዋል። ጥናቱ የሚካሄደው በቃለ መጠይቅ በሚተዳደር መጠይቅ ነው። ለእኛ የሚሰጡን መረጃ በራስ መተማመን ያለው እና ለዚህ የጥናት ዓላማ ብቻ ጥቅም ላይ የሚውል ነው። የኮድ ቁጥር እያንዳንዱን ተሳታፊ ያሳያል እና ምንም ስሞች አይጠቀሙም። የውጤቱ ሪፖርት ከታተመ የጠቅላላው ቡድን ጠቅለል ያለ መረጃ ብቻ ይታያል። ቃለመጠይቁ በፈቃደኝነት ነው - በቃለ መጠይቁ ወቅት በማንኛውም ጊዜ ለመሳተፍ ወይም ላለመሳተፍ ወይም ላለመቀበል መብት አለዎት። እርስዎ ወይም ማንኛውም የቤተሰብዎ አባል በሚቀበሏቸው አገልግሎቶች ላይ እምቢታዎ ምንም ዓይነት ተጽዕኖ አይኖረውም። ሆኖም የጥናት ክፍተቱን ለማሟላት የእርስዎ ተሳትፎ አስፈላጊ ነው። ማብራሪያ የሚያስፈልጋቸው ነገሮች ካሉ እባክዎን ማብራሪያዎችን ከመጠየቅ ወደኋላ አይበሉ።የግርጌ ማስታወሻ-መጠይቅ

እባክዎን በዚህ ጥናት ውስጥ መሳተፍ ይፈልጋሉ?

ሀ/ አዎ ፣ በጥናቱ ውስጥ መሳተፍ እፈልጋለሁ። (እባክዎን ወደ ቀጣዩ ገጽ ይሂዱ)

ለ/ አይ ፣ በጥናቱ ውስጥ መሳተፍ አልፈልግም።

| **ክፍል አንድꓽ- የጥናት ተሳታፊዎች ማህበረሰባዊ፣ኢኮኖሚያ ዲሞግራፊክ ባህሪዎች** | | | |
| --- | --- | --- | --- |
| 1 | | ዕድሜ (በዓመት) | -------------- |
| 2 | | የተሳታፊ ጾታ | ሀ. ወንድ ለ. ሴት |
| 3. | | የትምህርት ደረጃ | ሀ. ማንበብና መጻፍ አለመቻል  ለ. ማንበብ እና መጻፍ የሚችል  ሐ. የመጀመሪያ ደረጃ ትምህርት  መ. የሁለተኛ ደረጃ ትምህርት  ሠ. ኮሌጅ እና ከዚያ በላይ |
| 4 | | የሥራ ሁኔታዎ ምንድ ነው? | ሀ. የመንግስት ሰራተኛ  ለ. የግል ሠራተኛ  ሐ. ገበሬ  መ. ነጋዴ  ሠ. ተማሪ  ረ. ሌላ ፣ መጥቀስ ______ |
| 5 | | ሃይማኖትህ ምንድን ነው? | ሀ. ኦርቶዶክስ  ለ. ሙስሊም  ሐ. ፕሮቴስታንት  መ. ካቶሊክ  ሠ. ሌላ |
| 6 | | የጋብቻ ሁኔታዎ ምንድ ነው? | ሀ. ያላገባ  ለ. ያገባ  ሐ. የተፋታ(ች)  መ. መበለት(የሞተባት(በት) |
| 7 | | በቤተሰብዎ አማካይ ወርሃዊ ገቢ በኢትዮጵያ ብር | ሀ. ከ 1000 የኢትዮጵያ ብር ያነሰ  ለ. 1000-1999 የኢትዮጵያ ብር  ሐ. 2000-4000 የኢትዮጵያ ብር  መ. 4000 እና ከዚያ በላይ የኢትዮጵያ ብር |
| **8** | **ስንት የቤተሰብ አባላት አለዎት?** | | ሀ. አረት እና ከዚያ በተች  ለ.አረት በላይ |
| **ክፍል ሁለትꓽ- ከተጎዳኝ በሽታዎች ጋር የተዛመደ** | | | |
| 9 | | ማንኛውም ሥር የሰደደ በሽታ አለብዎት? | ሀ. አዎ ለ. አይደለም |
| 10 | | አዎ ከሆነ ፣ ምን ዓይነት ሥር የሰደደ በሽታ? | ሀ. የመተንፈሻ እና የሳንባ በሽታ  ለ. የካርዲዮቫስኩላር በሽታ  ሐ. የስኳር በሽታ  መ. ኤችአይቪ/ኤድስ  ሠ. ሌላ (ይግለጹ -------------------------------------------) |
| **ክፍል ሶስትꓽ- ስለ ኮቪድ-19 ዕውቀት** | | | |
| 11 | | ስለ ኮቪድ -19 ሰምተዋል | ሀ. አይደለም  ለ. አዎ |
| 12 | | ስለ አዲሱ ኮሮናቫይረስ ከየት ሰምተው ነበር? | ሀ. ሬዲዮ  ለ. ቴሌቪዥን  ሐ. መስተዳድር  መ. ማህበራዊ ሚዲያ  ሠ. ሌላ ይግለጹ________ |
| 13 | | በኮቪድ -19 ከተያዙ የት ነው የሚሄዱት? | ሀ. ወደ ሆስፒታል/ጤና ጣቢያ ይሂዱ  ለ. ባህላዊ ፈዋሽ  ሐ. ራስን ማከም  መ. ሌላ (ይግለጹ -------------------------------------------) |
| 14 | | የ ኮቪድ -19 ምልክቶች ምንድናቸው? | ከተሰጡት አማራጮች በምላሽዎ ላይ ክበብ ያድርጉ ፦  1. ደረቅ ሳል - ሀ. አዎ ለ. አይደለም ሐ. አላውቅም  2. ድካም - ሀ. አዎ ለ. አይደለም ሐ. አላውቅም  3. የጉሮሮ መቁሰል - ሀ. አዎ ለ. አይደለም ሐ. አላውቅም  4. የትንፋሽ እጥረት - ሀ. አዎ ለ. አይደለም ሐ. አላውቅም  5. ትኩሳት - ሀ1. አዎ ለ. አይደለም ሐ. አላውቅም  6. ራስ ምታት - ሀ. አዎ ለ. አይደለም ሐ. አላውቅም |
| 15 | | ኮሮናቫይረስ እንዴት ይተላለፋል? | ከተሰጡት አማራጮች በምላሽዎ ላይ ክበብ ያድርጉ ፦  1. በበሽታው ከተያዙ ሰዎች ጠብታዎች - ሀ. አዎ ለ. አይደለም ሐ. አላውቅም  2. የተበከለ ቁሳቁስ ከኮቪድ -19 በበሽታው ከተያዘ ሰው ነጠብጣብ  ሀ. አዎ ለ. አይ ሐ. አላውቅም |
| 16 | | የኮቪድ -19 ሕክምና ዘዴን ያውቃሉ? | ከተሰጡት አማራጮች በምላሽዎ ላይ ክበብ ያድርጉ ፦  1. ለኮቪድ -19 ውጤታማ የሆነ አንቲባዮቲክ ሕክምና የለም- ሀ. አዎ ለ. አይ ሐ. አላውቅም  2. የድጋፍ ሕክምና ከኮቪድ -19 ማገገምን ይረዳል- ሀ. አዎ ለ. አይ ሐ. አላውቅም  3. ኮቪድ -19 ከታከመ ይፈውሳል? ሀ. አዎ ለ. አይ ሐ. አላውቅም |
| 17 | | የኮቪድ -19 በሽታ መከላከያ ዘዴን ያውቃሉ? | ከተሰጡት አማራጮች በምላሽዎ ላይ ክበብ ያድርጉ ፦  1. የእጅ መታጠብ ፣ ቤት መቆየት ፣ የፊት ማስክ መጠቀም ፣ እጅ ከመጨባበጥ መቆጠብ እና የእጅ ማፅጃን መጠቀም የኮቪድ -19 ን ኢንፌክሽንን ይቀንሳል- ሀ. አዎ ለ. አይደለም ሐ. አላውቅም |
| **ክፍል አራትꓽ- ስለ የኮቪድ -19 አመለካከቶች እና ግንዛቤ** | | | |
| 18 | | የኮቪድ -19 የመከላከያ እርምጃዎች መከላከያ ነበሩ? | ሀ. አዎ  ለ. አይደለም |
| 19 | | አንዳንዶች በዙሪያዎ ቢቆሙ በራስ የመተማመን ስሜት ይሰማዎታል? | ሀ. አዎ  ለ. አይደለም |
| 20 | | የመንግስት ደንቦችን ማዳመጥ እና መከተል የኮቪድ -19 ስርጭትን ሊቀንስ ይችላል ብለው ያስባሉ? | ሀ. አዎ  ለ. አይደለም  ሐ. አላውቅም |
| **ክፍል አምስትꓽ- የመገልገያዎች ተገኝነት** | | | |
| 21 | | የፊት ጭንብል መዳረሻ (መግዛት ወይም መቀበል ይችላሉ)? | ሀ. አዎ  ለ. አይደለም |
| 22 | | አዎ ከሆነ ፣ ከየት? | ሀ. ፋርማሲ  ለ. የአከባቢ ሱቆች  ሐ. ከጤና ተቋማት  መ. ሌላ (ይግለጹ -------------------------------- |
| 23 | | አልኮሆል ወይም የእጅ ማጽጃ (ማጽጃ) አለዎት? | ሀ. አዎ  ለ. አይደለም |
| 24 | | በተደጋጋሚ እጅን ለመታጠብ የውሃ አቅርቦት | ሀ. አዎ  ለ. አይደለም |
| **ክፍል ስድስትꓽ- የመከላከያ እርምጃዎች** | | | |
| 25 | | የሚከተሉትን የኮቪድ -19 የመከላከያ እርምጃዎች በየቀኑ ተለማምደዋል? | ከተለዋጭ አማራጮች በመምረጥ በምላሽዎ ላይ ክበብ ያድርጉ  1. እጅዎን በሳሙና እና በውሃ በመደበኛነት ይታጠቡ -  ሀ. አዎ ለ. አይደለም  2. ሳሙና እና ውሃ በማይገኝበት ጊዜ በአልኮል ላይ የተመሠረተ ማጽጃ ይጠቀማል- ሀ. አዎ ለ. አይደለም  3. ምልክትን እና ምልክትን በሚያገኙበት ጊዜ ቤት መቆየት ሀ. አዎ  ለ. አይደለም  4. ከማንም ሰው ቢያንስ 2 ሜትር ርቀትዎን ይጠብቁ - ሀ. አዎ ለ. አይደለም  5. የሕዝብ ቦታዎች ላይ የፊት ማስክ ይጠቀማል - ሀ. አዎ ለ. አይደለም  6. የእጅ መንቀጥቀጥን ያስወግዱ ሀ. አዎ ለ. አይደለም |

የመረጃ ሰብሳቢ ስም:__________________ፍርማ__________ ቀን: __________

የተቆጣጣሪው ስም: _____________________________ ፍርማ። ___________________ ቀን: _________
