# Supplementary material for: Assessment of practice of Covid-19 preventive measures and associated factors among residents in Southern, Ethiopia
Source: PLoS One. 2021 Dec 10;16(12):e0261186. doi: 10.1371/journal.pone.0261186 (PMC8664224; doi:10.1371/journal.pone.0261186)
Supplement: S2 Questionnaire — (DOCX) [file pone.0261186.s002.docx]

**DILLA UNIVERSITY**

**COLLEGE OF MEDICINE AND HEALTH SCIENCE**

**DEPARTMENT OF PUBLIC HEALTH**

**Consent form**

Consent form that certify the respondents agreement before the interview assessment on practice of Covid 19 and associated factors in Wenago town, Gede’o zone, SNNPR, Ethiopia, 2020 GC.

Name of kebele ________________________

Questionnaire identification number _________________

Introduction: my name is _______________________ I am interviewing Wenago residents about practice of Covid 19 preventive measures and associated factors. You are selected to be one of the participants in the study. The study will be conducted through interviewer administered questionnaire. The information you give us is confident and will be used only for this study purpose. A code number will indentify every participant and no names will be used. If a report of the result is published only summarized information of the total group will appear. The interview is voluntary: you have the right to participate, or not to participate or refuse to do so at any time during the interview. Your refusal will not have any effect on services that you or any member of your family receives. However your participation is important to fulfill the study gap. If there are things that require clarification please don’t hesitate to ask the facilitators for clarification.

Do you mind participating in this study, please?

1. Yes, I want to participate in the study. (Please go to the next page)
2. No, I don't want to participate in the study.

| **Part I: socio-demographic characteristics** | | | | |
| --- | --- | --- | --- | --- |
| 1 | Age (in year) | | ----- | |
| 2 | Sex | | 1. Male 2. Female | |
| 3. | Educational status | | 1. Unable to read and write 2. Able to read and write 3. Primary education 4. High school education 5. College and above | |
| 4 | Occupational status | | 1. Gov’t employee 2. Private worker 3. Farmer 4. Merchant 5. Student 6. Other, mention______ | |
| 5 | Religion | | 1. Orthodox 2. Muslim 3. Protestant 4. Catholic | |
| 6 | Marital status | | 1. Single 2. Married 3. Divorced 4. Widowed | |
| 7 | Income of houshold | | 1. Less than 1000ETB 2. 1000-1999ETB 3. 2000-4000ETB 4. 4000 and above ETB | |
| 8 | Family size | | 1. < 4 members 2. >4 members | |
| **Part II: Comorbidity related** | | | | |
| 9 | . Do you have any chronic illness? | | 1. Yes 2. No | |
| 10 | If yes, what Type of chronic illness? | | 1. Respiratory and lung disease 2. Cardiovascular disease 3. Diabetes Melitus 4. HIV/AIDS 5. other (specify-------------------------------------------) | |
| **Part III: Knowledge towards COVID-19** | | | | |
| 11 | Have heard about Covid-19 | | | 1. No 2. Yes |
| 12 | Where did you hear about the new coronavirus from? | | | 1. Radio 2. TV 3. Governement 4. Social media 5. Other specify________ |
| 13 | 4.If you get infected by covid-19 where do you go? | | | 1. Go to hospital/Health center 2. Traditional healer 3. Self treatment 4. Other |
| 14 | What are the symptoms of COVID-19 | | | ***Circle on your response by choosing from the given alternatives:***   1. Dry cough: 1. Yes 2. No 3. I don’t know 2. Fatigue: 1. Yes 2. No 3. I don’t know 3. Sore throat: 1. Yes 2. No 3. I don’t know 4. Shortness of breath: 1. Yes 2. No 3. I don’t know 5. Fever: 1. Yes 2. No 3. I don’t know 6. Headache: 1. Yes 2. No 3. I don’t know |
| 15 | How does the coronavirus transmitted? | ***Circle on your response from the given alternatives:***   1. Droplets from infected people: 1. Yes 2. No 3. I don’t know 2. Contaminated material with droplet from Covid-19 infected person:   1. Yes 2. No 3. I don’t know | | |
| 16 | Do you know Covid-19 treatment method? | ***Circle on your response from the given alternatives:***   1. There is no effective antibiotic treatment available for Covid-19: 1. Yes 2. No 3. I don’t know 2. Does supportive therapy assissts recovery from Covid-19: 1. Yes 2. No 3. I don’t know 3. Is Covid-19 is curable if treated? : 1. Yes 2. No 3. I don’t know | | |
| 17 | Do you know prevention method of Covid-19? | ***Circle on your response from the given alternatives:***   1. Does handwashing, homestay,facemask use,avoiding hand shaking and applying hand sanitizer reduces Covid-19 infection: : 1. Yes 2. No 3. I don’t know | | |
| **Part IV: Attitudes and perception about COVID-19** | | | | |
| 18 | Do you believe Covid-19 preventive measures were protective? | 1. Yes 2. No | | |
| 19 | Do you feel insecure if some stands around you | 1. Yes 2. No | | |
| 20 | Do you think listening and following government regulation can reduce covid-19 transmission? | 1. Yes 2. No 3. Don´t know | | |
| **Part V: Availability of facilities** | | | | |
| 21 | Do you have access ( can you buy or recieve) to face mask? | 1. Yes 2. No | | |
| 22 | If yes, from where? | 1. Pharmacy 2. Local shops 3. from health institutions 4. other (specify------------------------------- | | |
| 23 | Do you have access alcohol or hand sanitizer? | 1. Yes 2. No | | |
| 24 | Do you have access water supply for frequent hand washing? | 1. Yes 2. No | | |
| **Part VI: Preventive measure practices** | | | | |
| 25 | Have practiced the following Covid-19 preventive measures daily? | ***Circle on your response by choosing from alternatives***   1. Wash your hand regularly with of soap and water: 1. Yes 2. No 2. Uses an alcohol-based sanitizer when soap and water are not available: 1. Yes 2. No 3. Home stay during get sign and symptom:1. Yes 2. No 4. Keep your distance of at least 2 meters from anyone: 1. Yes 2. No 5. Uses facemask at public places: 1. Yes 2. No 6. Avoid hand shaking:1. Yes 2. No | | |

Name of data Collector: ___________________________ Sign. _________________ Date: __________

Name of supervisor: _____________________________ Sign. ___________________Date: _________
